# Effects of packaging and ripeness on plantain flour characteristics during storage

**DOI:** 10.1002/fsn3.2946

**Published:** 2022-07-07

**Authors:** Fernande G. Honfo, Euloge C. Togbe, Matthijs Dekker, Noël Akissoe

**Affiliations:** ^1^ Laboratory of Food Sciences, Faculté des Sciences Agronomiques Université, d'Abomey‐Calavi Jéricho Cotonou Benin; ^2^ Laboratory of Plant Pathology, Faculté des Sciences Agronomiques Université, d'Abomey‐Calavi Jéricho Cotonou Benin; ^3^ Food Quality and Design Wageningen University AA Wageningen The Netherlands

**Keywords:** packaging materials, pasting viscosity, plantain flour, ripening stage, storage duration

## Abstract

The increasing implication of plantain flour in various food formulations calls for the need to evaluate the effects of ripening stage, packaging materials, and storage duration on its proximal composition and functional properties. For this study, plantain flours were produced from the cultivar *Alloga* at unripe and semiripe stage 3. They were stored both in transparent polyethylene bags and in an opaque aluminum foil. Physicochemical analyses and functional characterization of the plantain flour were performed on samples taken prior to storage and on monthly basis for 6 months during storage. Ash and carbohydrate contents decreased while the yellowness and redness increased with ripening. Pasting viscosity drastically decreased with ripening. During storage, significant differences in color and among most functional characteristics were observed as a consequence of both storage duration and packaging materials. Based on this research, flour from semiripe plantain could be recommended for use in formulations requiring low viscosity. Besides, it is suggested to store plantain flours in opaque containers to reduce the variability in its properties, thus maintaining its original quality.

## Graphical abstract

The study investigated on the proximal composition and functional properties of flours derived from plantain at two ripening stages, and packaged in different materials during storage. Pasting viscosity drastically decreased with ripening. During storage, significant differences in color and among most functional characteristics were observed as a consequence of both storage conditions and packaging materials. However, variations were less pronounced in flours packaged in opaque materials.

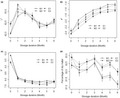

## 1 INTRODUCTION

Plantain is an important food crop in tropical and subtropical regions worldwide. In sub‐Saharan Africa, banana and plantain provide more than 25% of the energy needs of 70 million people (Daniells et al., 2011; FAOSTAT, 2019). Pulp from mature green plantain is rich in sugar (2%–31%), micronutrients, viz. potassium (440 mg/100 g), phosphorus (32 mg/100 g), and magnesium (32 mg/100 g), vitamin C (20 mg/100 g), and vitamin B (Kouadio, 2015; Robinson & Galan Sauco, 2010).

In plantain production areas, plantain pulps are consumed between the green and the yellow stages of ripening, after being boiled, pounded, fried, or roasted. Plantain pulp is also processed into flour for bakery and pastry. Besides, semiripe or ripe plantain can be used in infant food formulations as well as food for invalids who may have challenges in digesting carbohydrates (Yomeni et al., 2004). Ripening in plantain occurs once the fruit matures. During ripening, chlorophyll is degraded by the chlorophyllase enzyme to a colorless product (Fatemeh et al., 2012). In addition, carbohydrates are converted into simple sugars through α‐ and β‐amylases activity (Okezie et al., 2003). As such, semiripe or ripe plantain flour can be incorporated into food products requiring solubility, sweetness, and a small quantity of sugar.

However, the ripening stages of fruits and vegetables are determinants in the technical aspects of their processing. For example, banana pulp flour prepared using fruits at different ripening stages has been shown to behave differently during the manufacturing of food products such as cakes and extruded products (Gamlath, 2008; Yomeni et al., 2004). Moreover, the level of incorporation of plantain flour into food and bakery products is primarily governed by its physicochemical and functional properties (Fadimu et al., 2018). Many studies have characterized plantain flours in terms of their composition and functional properties and found that plantain flour is a good substitute for wheat flour and other similar starch flours in some food products (Aziz et al., 2014; Mepba et al., 2007; Onwuka & Onwuka, 2005). However, semiripe or ripe plantain flour is less known by consumers, although these products may offer a high content of carbohydrates due to the increase in sugar level during ripening.

Storage of plantain flour, for a few days up to several weeks, for further uses after processing, is a common practice. However, during storage, several factors could affect product quality. These factors are as follows: type of packaging materials used, storage environment (viz. temperature, light, oxygen, relative humidity), and storage duration (Gunstone, 2002; Obadina et al., 2016). Various changes caused by these factors, particularly regarding physicochemical reactions during storage, are reflected in many quality characteristics such as rancid flavor, changes in color, texture, and functional properties (Asikin et al., 2014). Therefore, adequate storage conditions are essential for maintaining the quality of plantain flour for a long duration.

While several researchers have focused on changes in plantain fruit during storage (Adi et al., 2019; Yang et al., 2009), specific studies on plantain flour storage are limited. Moreover, since plantain flour must be carefully handled and stored after production, it is necessary to test the different packaging materials used to determine which ones are suitable to preserve the flour quality during storage. In addition, information on physicochemical and functional changes in flours derived from plantain at different ripening stages, during storage, is necessary to predict flour quality for further uses. The present study, therefore, investigated the proximal composition and functional properties of flours derived from plantain at two ripening stages, and packaged in different materials during storage. The hypothesis underlying this objective was that physicochemical reactions cause changes in the functional quality of plantain flour during storage, depending on storage period, ripeness stage, and packaging materials used.

## 2 MATERIALS AND METHODS

### 2.1 Materials

Bunches of the local plantain cultivar “*Alloga”* were randomly harvested at the commercial stage (mature and unripe) in the farm named “Cité de Banane” located in the municipality of Zè (latitude: 6° 46′ 59.99“ N longitude: 2° 17’ 60.00” E), in Southern Benin. They were, then, divided into two parts: the first part was processed immediately into flour, whereas the second one was kept at room temperature (28 ± 2°C) until it reached the semiripening stage 3 (more green than yellow), according to the scale of Medlicott (1992), before being processed into flours. Two types of packaging materials with a capacity of 500 g and of rectangular shape (transparent polyethylene bag and opaque aluminum film bag) were purchased in a supermarket in Cotonou (Benin) for this purpose.

### 2.2 Methods

#### 2.2.1 Processing of flours

Flours were produced from unripe and semiripe plantain, following the procedure described below. After being washed and peeled, the pulp of the plantain was sliced (2–3 mm of thickness) and immersed in lemon water (1%–2%) to prevent enzymatic browning reaction (Gbadamosi & Oladeji, 2013). The slices were then oven dried at 55°C for 5–6 hours before being ground into powder, and sieved (Ndayambaje et al., 2019). Flours obtained were characterized for their proximal composition, pasting, and functional properties before being stored.

#### 2.2.2 Storage of unripe and semiripe plantain flour

Batches of 250 g of each type of flours were prepared, put in each type of packaging bag (transparent vs. black) in duplicated layer and then sealed properly with a sealing machine. They were then stored in a room at ambient conditions (temperature: 28 ± 2°C; relative humidity: 81% ± 3%) for 6 (6) months. A total of 24 samples were produced for each type of packaging: 12 for unripe plantain flours and 12 for semiripe plantain flours. Every month, two batches of each type of flour were taken from each packaging material to assess their dry matter content, color, and functional properties until the end of the storage period.

#### 2.2.3 Proximal composition assessment

Dry matter, ash, fat, and protein (N·x 6.25) contents were assessed by using the standard methods of analysis (AOAC, 2002). Carbohydrates content was determined by using the following Equation 1:

(1) %Carbohydrates=%Drymatter‐(%Protein+%Fat+%Ash).

Color characteristics were analyzed using a Hunter colorimeter (Hunter Associates Laboratory Inc. Reston. VA. USA), on the basis of L*(brightness; 100 = white, 0 = black), a* (+, red; −, green), and b* (+, yellow; −, blue) values.

#### 2.2.4 Functional characteristics

Pasting properties of the plantain flours were assessed using a Rapid Visco Analyzer, RVA (HAAKE, Viscotester iQ‐Air) (AOAC, 2002). For this, 3.5 g of plantain flour was dispersed in an aluminum canister containing 25 ml of distilled water. The mixture was thoroughly stirred, and the canister, fitted into the RVA, was subjected to the following temperature profile, under continuous stirring at 160 rpm: keeping at 50°C for 5 min, heating from 50°C to 95°C at 6°C min^−1^, keeping at 95°C for 5 min, cooling down to 50°C at 6°C min^−1^, and then keeping at 50°C for another 5 min. The viscosity profile indices recorded included: peak viscosity, setback viscosity, breakdown viscosity, final viscosity, pasting temperature, and peak time. Results were expressed in Poise (P) for all of the parameters, except for the pasting temperature expressed in °C, and peak time expressed in minutes.

The swelling power (SP) of the flours was determined at 80°C, while the water absorption capacity (WAC) was determined at ambient temperature, using the method developed by Beuchat (1977). The oil absorption capacity (OAC) of the flours was determined also using the method of Beuchat (1977). The method described by Yasumatsu et al. (1972) was used to assess the emulsion capacity of the flours. The apparent (bulk) density was determined following the method by Jones et al. (2000).

All these analyses were conducted in triplicate and the mean ± standard error values were reported.

### 2.3 Statistical analyses

Two statistical analyses were performed using the Statistical Package for Social Scientists (IBM SPSS) software (version 21.0). Firstly, the independent t‐test analysis was performed to assess differences between proximal composition and pasting characteristics of flours from the two ripening status. Secondly, an analysis of variance with three factors (state of ripening, packaging materials, and storage duration) was carried out to determine the effects of treatments and their interactions on dry matter, color, and functional characteristics of the flours. In case of significant differences, means were separated using the Tukey test (*p* < .05).

## 3 RESULTS AND DISCUSSION

### 3.1 Proximal composition and color characteristics of flours from unripe and semiripe plantain

Significant decreases in dry matter, ash, and carbohydrate contents of flours were observed, while, in contrast, a significant increase in fat content was found with the ripening status of the plantain (Table 1). Similar results were found by earlier findings that indicated changes in some biochemical parameters during the ripening process of plantain and some cooking bananas (Alkarkhi et al., 2011; Campuzano et al., 2018; Yomeni et al., 2004). The decrease in dry matter content (i.e., increase in moisture) might be attributed to an increase in sugar content in the pulp during ripening due to conversion of carbohydrates into simple sugars through enzymatic hydrolysis. This process generally modifies the osmotic pressure and facilitates migration of water from the peel to the pulp (Watharkar et al., 2021). Additionally, the conversion of carbohydrates into volatile aroma and the utilization of sugars in respiration during ripening may result in the reduction in carbohydrates content (Watharkar et al., 2021). In general, protein content increases during fruit ripening (Ayo‐Omogie et al., 2010); and this increase has been attributed to the possible enzyme synthesis and/or protein synthesis during ripening (Mamiro et al., 2007). During banana ripening, protein synthesis is, indeed, required for the development of flavor and texture, and for ethylene synthesis (Toledo et al., 2012). In the present study, a slight increase in protein content was observed, probably due to the stage of ripening chosen for the trial. In fact, significant increases may be expected from unripe to fully ripe fruit, thereby explaining the slight increase observed in the semiripe plantain. A low value of ash content was found in the flour from semiripe plantain compared to that in the unripe plantain flour, thereby witnessing the decrease in minerals content during ripening (Seraglio et al., 2018). However, the mean values of protein, fat, and ash contents recorded here are in line with those reported earlier for plantain flours by several authors (Kaur & Singh, 2005; Rodriguez‐Ambriz et al., 2008). A decrease in mineral content composition during ripening is a crucial physical event in the softening of banana fruits (Ahenkora et al., 1997).

**TABLE 1:** Proximal composition (% dry basis) and color parameters of unripe and semiripe plantain flours

Flours	Dry matter (%)	Ash (%)	Protein (g/100 g)	Fats (g/100 g)	Carbohydrates (g/100 g)	L*	a*	b*
Unripe plantain	93.36 ± 0.07^*^	2.52 ± 0.37	3.25 ± 0.38	0.29 ± 0.01	87.79 ± 0.13	82.87 ± 0.01	−2.10 ± 0.02	8.87 ± 0.06
Semiripe plantain	92.39 ± 0.12	2.19 ± 0.10	3.52 ± 0.00	0.36 ± 0.06	84.76 ± 0.09	82.03 ± 0.11	−2.95 ± 0.01	10.79 ± 0.04
P of ripening	0.001	0.032	NS	0.037	0.023	NS	0.001	0.021
P of packaging	0.001	–	–	–	–	NS	0.031	NS
P of storage duration	NS	–	–	–	–	NS	NS	NS
P of ripening × packaging	NS	–	–	–	–	NS	0.001	0.043
P of ripening × duration	0.035	–	–	–	–	0.001	0.001	0.001
P of packaging × duration	NS	–	–	–	–	0.012	0.012	NS
P of ripening × packaging × duration	NS	–	–	–	–	0.031	0.041	NS

Abbreviation: NS, nonsignificant.

* Mean ± standard error of mean.

As for flour color, significant increases were noticed for a* and b* values (Table 1), meaning that flours from unripe plantain were less red and less yellow than those from semiripe plantain. In that respect, Yang et al. (2009) observed that the color of the plantain pulp tends to increase in redness and yellowness during ripening; and they attributed this finding to the chlorophyll degradation that occurs during this period.

### 3.2 Functional characteristics of flours from unripe and semiripe plantain

#### 3.2.1 Pasting characteristics

Significant differences were observed for most pasting parameters evaluated among flours derived from plantain at the two stages of ripening (Table 2). Ripening tends to decrease the viscosity of the flours along with the viscosity profile. This observation is consistent with that by Campuzano et al. (2018) who noticed that the values, respectively, of the peak, final, setback, and breakdown viscosities in flours decreased with the ripening of plantain; and that decrease was the highest in flours from the third and fourth ripening stages. According to these authors, this decrease could be attributed to the degradation of starch into sugar, and to the enzymatic hydrolysis of carbohydrates. Thus, they stated that flours from the first and second ripening stages lead to firmer gels, with a higher tendency for retrogradation. Peak viscosity reflects the maximum viscosity developed during or immediately after cooking, and high value of this viscosity is referred to the strength of viscous pastes formed from gelatinization during processing (Adebowale et al., 2011; Fadimu et al., 2018). The differences recorded could be attributed to the sugar content that increases during ripening and that may tie up water molecules, making them unavailable for starch production (Ayo‐Omogie et al., 2010). Breakdown viscosity is linked to peak viscosity and often reflects the stability of the paste during cooking (Newport Scientific, 1998). Lower the breakdown viscosity, higher the ability of the sample to withstand heating and shear stress during cooking (Adebowale et al., 2011). In the present study, flours derived from semiripe plantain are expected to have the highest paste stability during cooking due to their lower value of breakdown viscosity (4.27 P vs. 16.09 P) (Table 2). The setback viscosity is linked to the retrogradation of starch molecules, and has an implication on the digestibility of the products derived from the flour (Sandhu et al., 2007). A high value of setback viscosity may, therefore, affect paste digestibility while its lower value could increase it (Sandhu et al., 2007). Lower values of the setback viscosity were found with flours from semiripe plantain compared to those from unripe plantain. In contrast, no significant differences were observed in peak times and pasting temperatures between the two types of flours.

**TABLE 2:** Pasting characteristics of unripe and semiripe plantain flours

Flours	Peak viscosity (Poise)	Breakdown viscosity (Poise)	Final viscosity (Poise)	Setback viscosity (Poise)	Peak time (min)	Pasting temperature (°C)
Unripe plantain	76.51 ± 0.33*	16.09 ± 1.00	81.85 ± 0.07	21.43 ± 0.61	12.68 ± 0.0	77.60 ± 0.42
Semiripe plantain	43.78 ± 2.07	4.27 ± 0.04	53.15 ± 1.03	13.05 ± 0.49	13.14 ± 0.15	76.75 ± 3.04
*p*‐value	.000	.000	.000	.000	.023	.063

* Mean ± standard error of mean.

#### 3.2.2 Other functional characteristics of flours from unripe and semiripe plantain

Ripening had a significant effect on most functional characteristics investigated, except on the bulk density (Table 3). Water and oil absorption capacities, swelling power, as well as emulsion capacity decreased significantly with ripening. A similar trend has been reported by Ayo‐Omogie et al. (2010), who found that the decrease was more pronounced at the ripening stages 5 to 7. Most of these decreases were attributed to the conversion of starch into simple sugars (Fagbemi, 1999), which are known to inhibit starch hydration, thereby, leading to a reduction in water absorption by flours. Moreover, Tortoe et al. (2009), associated the higher values of water absorption to the higher starch content in flour from unripe plantain whose complex molecules would request more water during hydration than sugar molecules. However, high water absorption and swelling capacities have both economic and culinary advantages, since flour from unripe plantain appears to be better suited for the production of pastry and baking foods.

**TABLE 3:** Other functional characteristics of unripe and semiripe plantain flours

Flours	Water absorption capacity (%)	Oil absorption capacity (%)	Swelling power at 80°C (%)	Bulk density (g/cm^3^)	Emulsion capacity (%)
Unripe plantain	159.96 ± 0.49*	91.38 ± 0.78	8.82 ± 0.06	0.87 ± 0.0	49.04 ± 1.36
Semiripe plantain	154.43 ± 0.46	83.42 ± 0.75	7.36 ± 0.17	0.91 ± 0.01	43.15 ± 1.20
P of ripening	0.012	0.023	0.001	NS	0.021
P of packaging	0.023	0.037	0.001	NS	0.041
P of storage duration	NS	0.001	0.001	NS	0.031
P of ripening × packaging	0.023	NS	0.001	NS	0.023
P of ripening × duration	0.001	0.001	0.001	NS	0.001
P of packaging × duration	0.037	0.001	0.001	NS	0.041
P of ripening × packaging × duration	0.001	0.023	0.001	NS	0.037

Abbreviation: NS, nonsignificant.

* Mean ± standard error of mean.

Values of bulk density did not differ between the two types of flours (Table 3). Bulk density is an important characteristic in determining the choice of the packaging materials to be used during handling and preservation of food (Ixtaina et al., 2008). Its low value may constitute an advantage in the formulation of infants' foods in which high nutrient content and low bulk density are rather desired (Abass et al., 2009).

Emulsion capacity (EC) is generally related to the volume of oil that can be emulsified per gram of protein (Fagbemi, 1999). A significantly lower EC value was observed in flour from semiripe plantain compared to that from the unripe one (Table 3). This may probably be due to differences in protein contents between the two types of flours. Similar results have been reported in previous studies that pointed out significant decreases in the emulsion capacity (EC) from stages 1 to 7 of plantain ripening (Ayo‐Omogie et al., 2010; Fagbemi, 1999). In addition, significant interactions between the ripening stage and the storage duration were recorded on EC whose values increased during flour storage (Table 3).

### 3.3 Effects of packaging on color and dry matter of flours during storage

**FIGURE 1:**
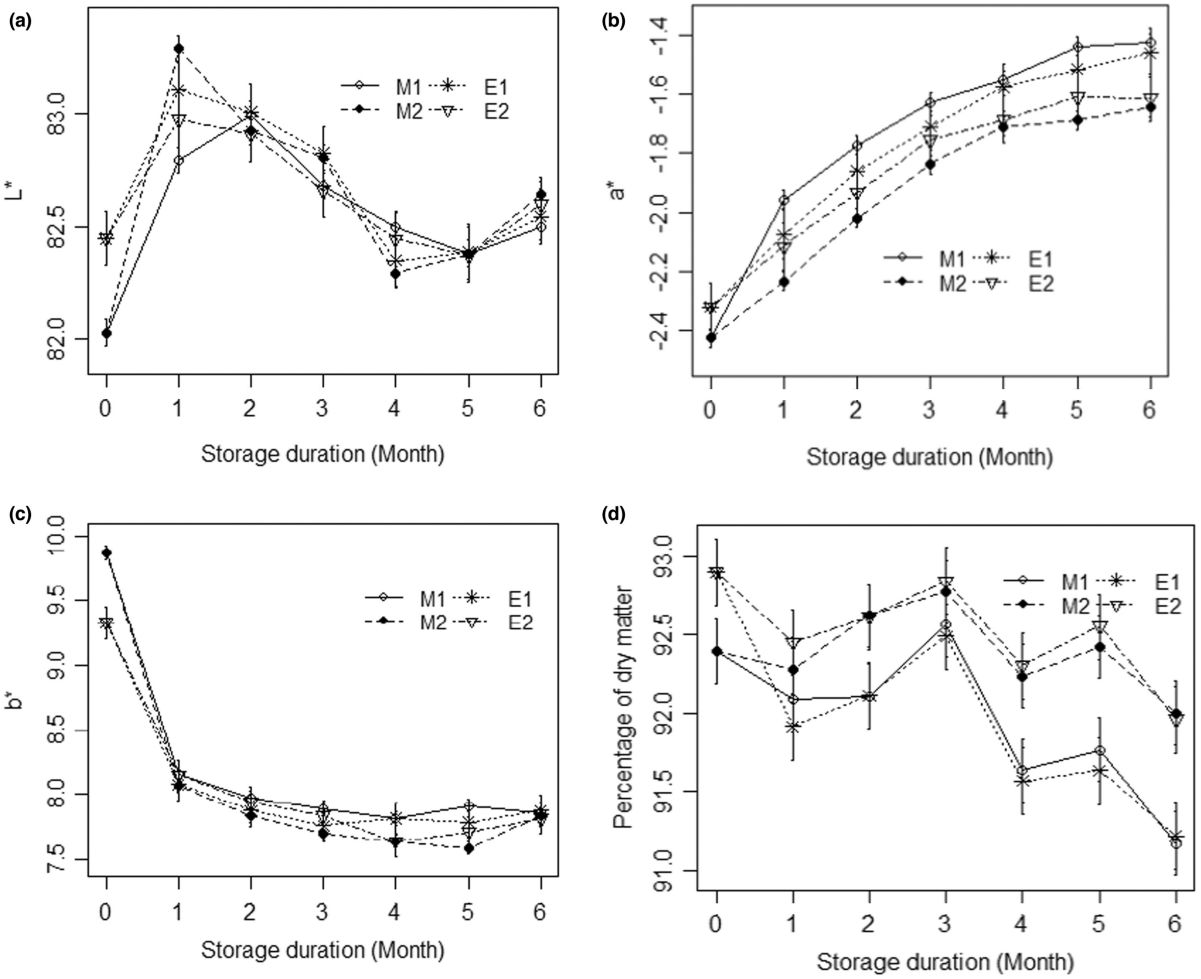
Effects of packaging on color parameters of flours during 6 months of storage. (a) Dry matter; (b) Luminance L*; (c) Redness (a*); (d) Yellowness (b*); M1: Flour from unripe plantain; M2: Flour from semiripe plantain; E1: Transparent packaging; E2: Opaque packaging

Between the two types of flour packaged in the different packaging materials, fluctuations of luminance (L*) values followed similar trend throughout the storage period (Figure 1a). Packaging materials and storage duration had, therefore, no significant effects on L* values; however, L* values were significantly affected by their interaction (Table 1). Redness (a*) of flours was significantly influenced by packaging materials during storage period, as its values significantly increased in the two types of flours, regardless of the packaging materials used (Table 1; Figure 1b). In contrast, a gradual decrease in the b* values for the two types of flours was observed during the storage period (Figure 1c). However, the decrease did not significantly differ compared to the values recorded before the storage (Table 1), whereas significant interactive effects of both ripening stage and storage duration were observed on all the color characteristics targeted (Table 1). The varying behavior observed in the color characteristics during storage is certainly due mainly to the chemical reactions that occurred in the flours, especially by the oxidation and hydrolysis reactions of fatty acid (Jiang et al., 2021), and also the Maillard reaction (Karathanos et al., 2006).

During storage, the dry matter content of flours was significantly affected by the packaging materials (Figure 1d). The interactions between storage duration and ripening stage also had significant effects on the dry matter through a reduction in its content after 6 months of storage, witnessing the increasing moisture content of flours (Table 1).

Comparison between packaging materials revealed a more pronounced decrease in dry matter content in flours packaged in the transparent material probably due to its texture (i.e., particle composition). However, the increase in moisture during storage can mostly be linked to the hygroscopic nature of flours, as reported by Pragati et al. (2014). According to these authors, hygroscopicity is due to the attainment of equilibrium between the product and the surrounding environment at a particular relative humidity and temperature conditions. However, dry matter contents recorded in all flours at the end of the storage period (91–93%) were comparable to the threshold tolerated (90%) for a long‐term storage at ambient temperatures (27–29°C) (Sugri et al., 2017).

### 3.4 Effects of packaging on functional characteristics of flours during storage

**FIGURE 2:**
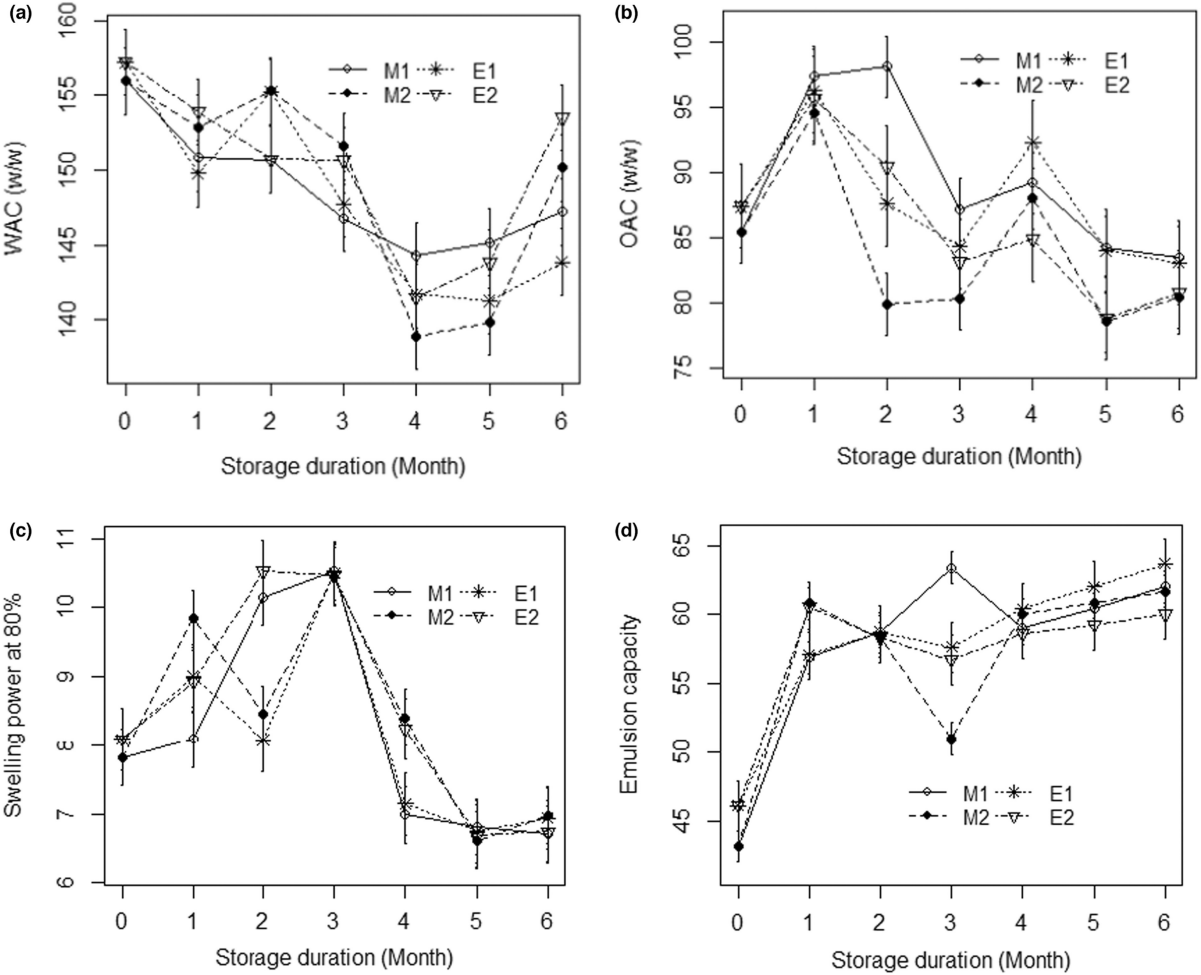
Effects of packaging on functional properties of flours during 6 months of storage. (a) Water absorption capacity; (b) Oil absorption capacity; (c) Swelling power at 80%; (d) Emulsion capacity; M1: Flour from unripe plantain; M2: Flour from semiripe plantain; E1: Transparent packaging; E2: Opaque packaging

Water absorption capacity (WAC) of flours fluctuated during storage, following a decreasing trend until 5 months (Figure 2a). However, the fluctuation was most pronounced in flours packaged in transparent plastic (145%) versus for opaque packaging (155%), thereby resulting in a significant difference between the values recorded for this parameter (Table 3). According to Pragati et al. (2014), the decrease in WAC values during flour storage is mainly due to the increase in moisture content (i.e., moisture uptake decrease) caused by the establishment of equilibrium moisture content of the flour during storage. However, Awoyale et al. (2016) attributed the decrease in WAC values to the chemical composition of flour, coupled with sunlight resistance of the packaging materials used. In fact, opaque packaging materials resist more to sunlight than transparent packaging materials. In addition, the rate of water absorption increased gradually from polyvinyl chloride packaging to polyethylene packaging, and then to polypropylene packaging materials (Awoyale et al., 2016). However, interactive actions of factors taken two by two as well as the interaction of the three factors (i.e., ripening × packaging × storage duration) altogether had significant effects on the WAC values of flours (Table 3); thereby, suggesting that the impact of storage conditions on WAC of plantain flours can well be altered by the ripening stage of the plantain.

Packaging material as well as its interaction with the storage duration had a significant effect on the oil absorption capacity (OAC) of the flours (Table 3). OAC values decreased from 87% to 83% in transparent packaging, and from 87% to 79% in opaque packaging, at the end of the storage period (Figure 2b). Such a decrease in OAC values during storage has been observed in earlier studies and has been associated with the reduced ability of the flour to entrap fat to the polar end of its protein chain due to the probable reduction in protein content during storage (Abeshu et al., 2016; Awoyale et al., 2020). However, the OAC of flour, in the present study, was influenced by the interaction of the three factors (Table 3).

The results of this study showed significant differences in the swelling power (SP) of flours at 80°C in relation to the packaging materials, the ripening stage, and the storage duration (Table 3). In fact, during the first 3 months of storage, a significant fluctuation of SP was similarly observed for the two types of flours (Figure 2c). After 6 months of storage, the decrease in SP values, in both packaging materials, was significantly pronounced compared to the values obtained before storage. However, since SP is the ability of flour starch to adsorb water to the extent that it causes the starch granules size to increase, its decrease toward the end of the storage period could be explained by the decrease in the flour's ability to adsorb water after a certain storage time. A long storage period would, therefore, have a reducing effect on the water absorption capacity (WAC) of the flour.

The emulsion capacity (EC) was significantly affected by the packaging materials during storage with significant interactions among the three factors, either taken two by two or altogether (Table 3). Overall, an increase in EC values was recorded in both packaging systems during storage, with a greater increase in flours packaged in the transparent materials (Figure 2d). A similar trend of increase in EC values was reported by Awoyale et al. (2016) in plantain flours stored in different materials. However, according to Liu et al. (2008), fluctuations of EC values during storage of flours were more complex and could be affected by many factors including particles size, protein content, packaging materials, storage conditions, etc. In a recent study, Awoyale et al. (2020) revealed that functional and pasting properties of unripe plantain flours were affected both by packaging materials and storage periods; thereby resulting in decreasing the viscosity aptitude of the flours. With regards to these findings, it appears that storage duration and packaging materials can affect the viscosity properties of flours from both unripe and semiripe plantain.

## 4 CONCLUSION

This study showed that the ripening stage of plantain modified most of the proximal composition, and functional properties of flours derived from it. While pasting viscosity drastically decreased with the ripening, packaging materials and storage duration significantly influenced dry matter content, colors, and most of the functional parameters of plantain flours, regardless of the ripening stage of the plantain used. Changes were, however, less pronounced in flours packaged in opaque materials compared with transparent materials. It derives from these results that flour from semiripe plantain could be used in formulations requiring low viscosity such as infant food formula. It can also be suggested to store plantain flours in opaque materials to reduce fluctuations in its functional properties and color parameters. For further research, FTIR or X‐ray analysis is advised for an in‐depth analysis on the physicochemical changes that generally occur during plantain storage.

## Data Availability

Research data are not shared. Due to privacy clauses between financial partners and our structure, we do not have permission to share the raw data that support the findings of this study. However, some data can be requested for a specific reason from the corresponding author.
